# Bendamustine vs. fludarabine/cyclophosphamide lymphodepletion prior to BCMA CAR-T cell therapy in multiple myeloma

**DOI:** 10.1038/s41408-023-00929-0

**Published:** 2023-10-13

**Authors:** Surbhi Sidana, Hitomi Hosoya, Alexandria Jensen, Lawrence Liu, Anmol Goyal, Vanna Hovanky, Bita Sahaf, Sushma Bharadwaj, Theresa Latchford, Sally Arai, Sheryl Leahy, Matthew Mei, Lihua E. Budde, Lori S. Muffly, Matthew J. Frank, Saurabh Dahiya, Myo Htut, David Miklos, Murali Janakiram

**Affiliations:** 1 Division of Blood and Marrow Transplantation and Cellular Therapy, Stanford University School of Medicine, Stanford, CA USA; 2 Quantitative Sciences Unit, Stanford University School of Medicine, Stanford, CA USA; 3 City of Hope Cancer Center, Duarte, CA USA; 4 Cancer Correlative Science Unit, Stanford University, Stanford, CA USA

**Keywords:** Cancer, Haematological cancer

## Dear Editor,

Two BCMA CAR-T constructs are now approved for relapsed multiple myeloma, idecabtagene vicleucel (ide-cel) and ciltacabtagene autoleucel (cilta-cel) [1–3]. Lymphodepletion prior to CAR-T therapy creates a favorable immune and cytokine milieu for CAR-T expansion, as well as direct cytotoxic impact, and is associated with improved efficacy [4–7]. It has been shown that combination of fludarabine and cyclophosphamide (Flu/Cy) is superior to cyclophosphamide lymphodepletion alone in B cell malignancies, and is routinely used prior to investigational or standard-of-care BCMA CAR-T therapy [5], although the initial study of LCARB38M (subsequently cilta-cel) used cyclophosphamide alone [8]. In 2022, we experienced a worldwide shortage of fludarabine [9], necessitating the adoption of alternative lymphodepletion. Here we report on the safety and efficacy of standard of care BCMA CAR-T therapy with bendamustine lymphodepletion from two centers and compare outcomes with Flu/Cy lymphodepletion.

We included consecutive patients receiving standard of care BCMA CAR-T therapy (ide-cel and cilta-cel) at two centers, Stanford University and City of Hope, from May 2021 to December 2022, with follow-up data collected through June 30, 2023. A total of 56 patients were identified, with 14 receiving bendamustine and 42 receiving Flu/Cy. The median follow-up in the bendamustine cohort was 10.4 months while for Flu/Cy cohort was 12.8 months. All surviving patients had at least 6 months of follow-up. Bendamustine was administered at 90 mg/m2 on days -4, -3 prior to CAR-T therapy, with day of CAR-T infusion considered as day 0. Three patients received bendamustine on days -5 and -4 due to change in institutional protocol. For patients receiving Flu/Cy, cyclophosphamide 300 mg/m^2^ and fludarabine up to 30 mg/m^2^ were administered on days -5, -4, and -3 prior to CAR-T infusion. Fludarabine dose was adjusted based on creatinine clearance per institutional protocols. Cytokine release syndrome (CRS) and neurotoxicity/immune effector cell-associated neurotoxicity syndrome (ICANS) were assessed based on ASTCT criteria [10, 11]. Hematologic toxicity was graded based on the Common Terminology Criteria for Adverse Events (CTCAE), version 5.0. Response assessment was based on the International Myeloma Working Group (IMWG) Criteria per investigator evaluation [12]. CAR-T expansion via flow cytometry was assessed in patients treated at Stanford as described under supplemental methods Table S1.

Statistical methods are described in detail in supplementary data. A covariate balancing propensity score weighting scheme was used to balance patient characteristics between treatment groups by weighting each patient in the analytic dataset by the inverse probability of receiving their actual exposure (IPTW). Sensitivity analysis for safety and efficacy by type of CAR-T therapy (ide-cel and cilta-cel) was also done to account for its potential confounding effect. Response rates in were compared using a weighted multivariable logistic regression comparing bendamustine vs Flu/Cy lymphodepletion in the IPTW sample after adjusting for CAR-T type. Survival outcomes were estimated by Kaplan Meier method and compared using weighted Cox proportional hazards regression in the IPTW sample after adjusting for CAR-T type.

There was no difference in baseline characteristics of patients receiving bendamustine vs Flu/Cy lymphodepletion including age, sex, race, presence of extramedullary disease (64 vs 57%), high-risk cytogenetics (43 vs 60%; defined as presence of del17p, t(4;14),t(14;16) and/or gain 1q), median prior lines of therapy (5 vs 6), triple-class (79 vs 86%) or penta-class (50% each) refractory disease and receipt of bridging chemotherapy (72 vs 83%), respectively. (Supplementary Table S2) In the bendamustine group, 6 (43%) patients received ide-cel and 8 (57%) patients received cilta-cel. In the Flu/Cy group, 30 (71%) patients received ide-cel and 12 (29%) patients received cilta-cel (*p* = 0.11). This difference is explained by earlier approval of ide-cel in 2021, when availability of fludarabine was not limited. Fludarabine shortage in mid 2022 overlapped with approval of standard of care cilta-cel in Feb 2022.

**Fig. 1:**
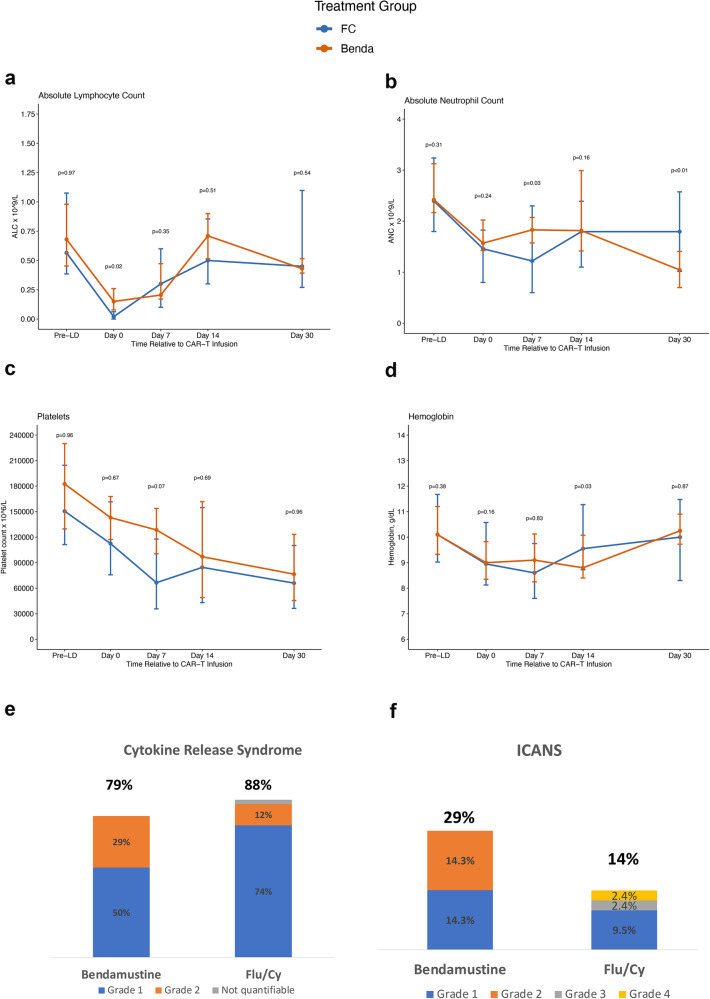
Safety outcomes after bendamustine and fludarabine/cyclophosphamide lymphodepletion chemotherapy in patients receiving BCMA directed CAR-T cell therapy: Kinetics of change in hematologic parameters and cytokine release syndrome (CRS) and immune effector cell associated neurotoxicity (ICANS). Cell counts following bendamustine (*n* = 14) and fludarabine/cyclophosphamide (Flu/Cy; *n* = 42) lymphodepletion chemotherapy in patients receiving BCMA directed CAR-T cell therapy: **a** Absolute lymphocyte count (ALC), **b** absolute neutrophil count (ANC), **c** platelet count, and **d** hemoglobin, **e** cytokine release syndrome and **f** immune effector cell associated neurotoxicity (ICANS). The line graphs for hematologic parameters **a**–**d** show median values with interquartile range at pre-lymphodepletion, days 0, 7, 14, and 30. Data were available in all patients at all timepoints shown, except ALC and ANC data was missing in one patient at day 0 and 7 in the fludarabine/cyclophosphamide group.

Bendamustine was effective at achieving lymphodepletion, with median ALC of 0.15 × 10^9^/L on day of infusion and nadir median ALC of 0.10 × 10^9^/L occurring 1.5 days following CAR-T infusion (Fig. 1a, Supplementary Table S3). Comparatively, the day of infusion ALC (0.02 × 10^9^/L, *p* = 0.02) and nadir ALC (0.01 × 10^9^/L, *p* < 0.01) were lower in the Flu/Cy group, with nadir in this group occurring 1 day after CAR-T infusion. Lymphocyte recovery occurred at a similar trajectory in both cohorts, and lymphocyte counts were comparable at all timepoints until day 90 (Supplementary Table S3). This finding is comparable to observations with bendamustine lymphodepletion prior to CD19 CAR-T therapies, where nadir ALC has been lower with Flu/Cy compared to bendamustine, but there has been no other observed difference in lymphocyte recovery [13, 14]. Patients in the Flu/Cy group had lower nadir ANC in the first 30 days following CAR-T infusion and at day 7 following infusion compared to the bendamustine group. However, at day 30 bendamustine cohort had a lower ANC than the Flu/Cy cohort with the point estimates for ANC being 1.05 vs 1.80 × 10^9^/L (*P* < 0.01), respectively. There were no significant differences in neutrophil count at other time points till day 90. There was no significant difference in platelet counts amongst the two groups, though platelets were numerically lower in the Flu/Cy group at certain timepoints. (Fig. 1; Supplementary Table S3)

**Fig. 2:**
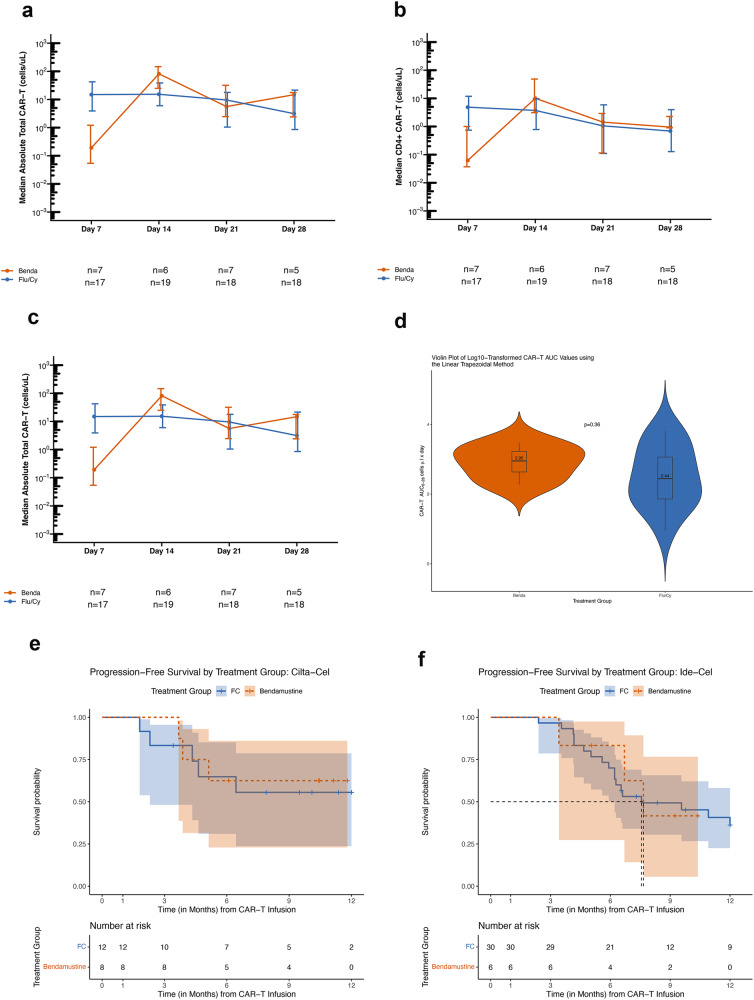
BCMA directed CAR-T expansion and efficacy after bendamustine and fludarabine/cyclophosphamide lymphodepletion. **a** Total CAR-T expansion **b** CD4 + CAR-T cell expansion **c** CD8 + CAR-T cell expansion. **d** Violin Plot of Log transformed CAR-T AUC values for CAR-T expansion in first month post CAR-T. **e** progression free survival in patients receiving cilta-cel, and **f** progression free survival in patients receiving ide-cel.

CAR-T expansion by flow cytometry was evaluated on patients treated at Stanford as described under supplemental methods. Figure 2a–d shows CAR-T expansion at different time points in the first month post infusion for total CAR-T+ cells, as well as CD4 and CD8 CAR-T cell subsets. There was no difference in CAR-T expansion by flow cytometry between the Flu/Cy (*n* = 22) and bendamustine (n = 8) patients over the first 30 days as measured by area under the curve (p = 0.36; Fig. 2d). We also compared median CAR-T cell expansion amongst patients in the two groups at individual timepoints and there were no statistically significant differences in CAR-T expansion on days 14 (*p* = 0.21) or 21 (*p* = 0.43) but there was a significant difference on day 7 (*p* < 0.01). (Fig. 2a–c) Given small sample size, we were not able to analyze the CAR-T expansion data separately for ide-cel and cilta-cel.

Safety and efficacy were comparable across the two cohorts as shown in Fig. 1e, f, Tables S4 and S5. Rates of CRS and ICANS were similar across the two groups. CRS was seen in 79% patients in the bendamustine cohort, with 29% being grade 2 or higher and 86% in the Flu/Cy cohort, with 14% being grade 2 or higher. There were no grade 3 or higher CRS events in either treatment group. There was no difference in median time to onset of CRS. The median time to CRS onset for ide-cel was 0.5 to 1 day and for cilta-cel was 7 to 8 days. ICANS occurred in 29 vs. 14% of patients receiving bendamustine vs Flu/Cy, respectively, *p* = 0.09. There were 2 grade ≥ 3 ICANS events, both of which occurred in the Flu/Cy group. Proportions of tocilizumab use, steroid use, ICU admission and hospital readmission were comparable between the two groups. Proportions of patients with neutropenic fever were also comparable, though numerically lower in the bendamustine group. (7 vs 36%, *p* = 0.37). Infections (21 vs 35%), G-CSF use (71 vs 93%), and IVIG use (33 vs 29%) were also similar. Patients receiving bendamustine had lower transfusion requirements, with fewer patients needing platelet transfusions (0 vs 33%, *p* < 0.01), though no statistical difference was noted in terms of packed RBC transfusions (7 vs 43%, *p* = 0.15).

Response rates were comparable in the two groups on multivariable logistic regression in the IPTW sample after adjusting for CAR-T type (Supplementary Table S5). Four patients had non-measurable disease and could not be evaluated for response, all within the Flu/Cy group. The overall response rate (ORR) for patients receiving cilta-cel (Bendamustine *n* = 8, Flu/Cy *n* = 12) was 88 vs 83% and for patients receiving ide-cel (Bendamustine *n* = 6, Flu/Cy *n* = 26) was 67 vs 88% (*p* = 0.60), respectively for bendamustine vs Flu/Cy lymphodepletion. The complete response (CR) rate for patients receiving cilta-cel was 50% in both groups and for patients receiving ide-cel was 67 vs 38%, *p* = 0.52, respectively for bendamustine vs Flu/Cy lymphodepletion. Progression free survival (PFS; Fig. 2e, f) and overall survival (OS) (Supplementary Figure S2) were similar in patients receiving bendamustine or Flu/Cy lymphodepletion in both the cilta-cel cohort and the ide-cel cohort on multivariable Cox proportional hazards analysis in the IPTW sample after controlling for CAR-T type. Median PFS was not reached in cilta-cel treated patients, with 6-month PFS estimate for bendamustine vs Flu/Cy being: 63 vs 65%; while in the ide-cel cohort, the median PFS was 7.7 vs 7.6 months, respectively (*p* = 0.99) (Fig. 2e, f). Median OS was not reached in both cilta-cel and ide-cel treated patients. 6-month OS estimate for cilta-cel treated patients in the bendamustine vs Flu/Cy cohort was 86 vs 73% and for ide-cel treated patients was 100 vs 87%, respectively; *p* = 0.11. (Supplementary Fig. S2, Table S5)

In this first report on the use of bendamustine lymphodepletion with BCMA CAR-T therapy, we observed that bendamustine is associated with a similar safety and efficacy profile as Flu/Cy lymphodepletion. In line with prior reports [14], bendamustine lymphodepletion chemotherapy was effective at decreasing the ALC, and the trajectory of lymphocyte count recovery was similar in the two groups, though ALC nadir was higher compared with Flu/Cy. Bendamustine lymphodepletion was associated with a higher ANC nadir and fewer transfusions when compared with Flu/Cy. The higher ANC nadir and lower transfusion requirement in the bendamustine group are comparable to prior reports [13, 14]. However, there was a delayed recovery of ANC when compared to Flu/Cy, although rates of infections were comparable. Importantly, rates of CRS and ICANS, as well as efficacy outcomes (response rates, PFS, and OS) appear comparable. While our findings align with most published data in hematologic malignancies, it contrasts with one report where bendamustine as single agent lymphodepletion in 5 patients prior to anti-CD30 CAR-T therapy for Hodgkin’s disease had a less favorable cytokine profile and no responses were seen in any of these patients [15]. It is difficult to compare this data with our study, given the small patient numbers and different types of hematologic malignancy in patients treated with anti-CD30 CAR-T therapy. Given the recent occurrence of fludarabine shortage necessitating the use of bendamustine, the long-term impact of bendamustine lymphodepletion on the safety and efficacy of BCMA-directed CAR-T therapy remains to be determined. In summary, bendamustine shows a similar safety and efficacy profile as Flu/Cy prior to BCMA CAR-T therapy although with certain differences.

### Supplementary information

Supplementary Data
