# Supplementary material for: Bendamustine vs. fludarabine/cyclophosphamide lymphodepletion prior to BCMA CAR-T cell therapy in multiple myeloma
Source: Blood Cancer J. 2023 Oct 13;13(1):158. doi: 10.1038/s41408-023-00929-0 (PMC10576036; doi:10.1038/s41408-023-00929-0)

Supplementary Data

Supplemental Methods:

Each site obtained their own IRB approval. At Stanford, patients were enrolled onto a biobank/data repository protocol prospectively and provided consent. At City of Hope, patients were identified retrospectively, and consent was waived.

1. Analysis: Descriptive statistics were used to characterize patients by treatment group. Univariate analysis was done using Chi-Square and Fischer's exact tests for categorical variables and Wilcoxon Rank Sum/Kruskal Wallis test for continuous variables for Supplementary Table 1. Statistical analyses for clinical and laboratory data were run R (version 4.3.0).

Propensity score weighting using inverse probability of treatment weighting (IPTW) was used to adjust for confounding variables amongst the two lymphodepletion groups (Bendamustine and Flu/Cy). The following baseline characteristics were included in the IPTW weighting: age, high risk cytogenetics by FISH (high-risk included gain 1q), presence of extramedullary disease, ISS stage, penta-refractory status, use of bridging therapy, as well as the following lab values at start of lymphodepletion therapy: absolute neutrophil count, absolute lymphocyte count and platelet count. Subsequently we ran a sensitivity analysis by including type of CAR-T received as a confounding variable in this propensity weighted cohort. Covariate balance by IPTW was assessed using the standardized mean difference (SMD), with a SMD of < 0.1 considered to indicate a good balance of that variable between treatment groups. As can be seen in Figure S1 below, before balancing 8 of the 9 variables included in the weighting scheme had an SMD > 0.1, with 5 having an SMD > 0.2. After balancing, all variables had an SMD < 0.1. Therefore, the covariates have been balanced between the two groups sufficiently. *(Reference: Austin PC. Stat Med. 2009 Nov 10;28(25):3083-107. doi: 10.1002/sim.3697. PMID: 19757444)*

All p-values, except those in supplementary **Table 2 showing** baseline characteristics, reflect adjusted p-values from propensity score weighting.

Figure S1: Propensity Score Weighting by Inverse Probability of Treatment Weighting amongst patients receiving Bendamustine vs Flu/Cy Lymphodepletion Prior to BCMA Directed CAR-T Therapy

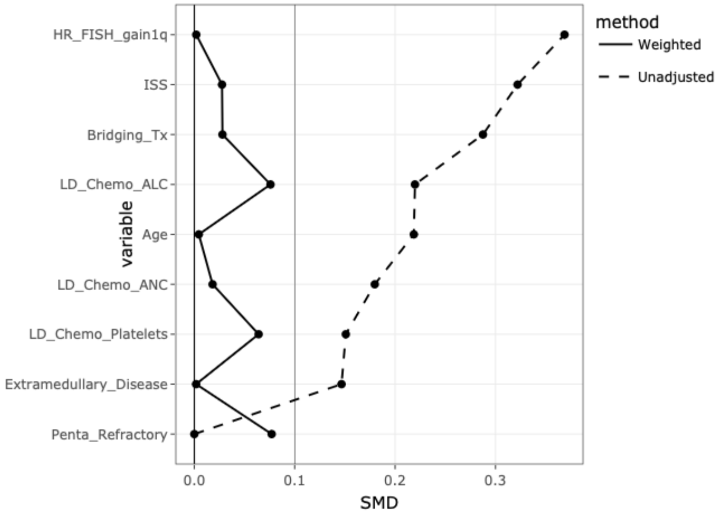

2. Flow Cytometry Assay for CAR-T Expansion

CAR-T expansion by flow cytometry was assessed on peripheral blood samples for patients treated at one center (Stanford University) at day 7 ($\pm$ 2), day 14 ($\pm$ 4), day 21 ($\pm$ 4) and day 28 ($\pm$ 4) following CAR-T cell therapy.

A High Dimensional (Hi-D) Immuno-phenoptyping flow cytometry panel was designed for immune profiling of CART- and target B cells in real time. PBMC were isolated from fresh whole blood by gradienrt centrifugation on ficoll (Ficoll paque Plus, GE Healthcare, SigmaAldrich). Two-5 million PBMC were stained with fixable Live/Dead aqua (Invitrogen) amine-reactive viability stain. Cells were then preincubate with Fc block (trustain, Biolegend) for 5 min, then stained at room temperature with the following (Table S1 fluorochrome conjugated mAb in an 10-color, 12-parameter staining combination. Tranduced T cells were used as positive control included in daily staining experiments. Stained and fixed cells were acquired on a LSR (BD BioSciences) four-laser analyzer (Blue-488 nm, Violet-405 nm, Red-640nm, Green 532 lasers and 21 parameters). At least, 10^6 cells were acquired unless restricted by the number of cells isolated from 8 ml of whole blood. The assay limit of detection for cells calculated as 1 in 10^4 of total acquired PBMCs. Absolute CAR T cell numbers were calculated by multiplying the percentages of CAR T cells among lymphocytes by the absolute lymphocyte count obtained on the same day

Table S1: Flow Cytometry Panel for CAR-T cell detection

Antigen	Fluorochrome	Supplier	Part number
CD3	FITC	BioLegend	300406
Human BCMA	PE	ProMab	BCA-HP2H2
CD14	PE-CY7	BioLegend	367112
CD8	PerCP Cy5.5	BD Pharmingen	565310
CD56	APC.Fire-750	BioLegend	362554
L/D cell marker		Invitrogen	L-34965
CD22	BV421	BioLegend	302524
CD20	BV605	BioLegend	302334
CD4	BV711	BioLegend	300558
CD45	BV785	BioLegend	368528

3. CAR-T Expansion Analysis

Absolute CAR-T cells were plotted as log-transformed values over time, as shown in Figure 2 a - 2c, for total CAR-T cells, as well as CD4+ CAR-T cells and CD8+ CAR-T cells. Mann-Whitney U tests were conducted to compare total CAR-T expansion at days 7, 14, and 21, respectively, between treatment groups.

Areas under the curve (AUCs) were calculated for CAR-T expansion in the 28 days following date of infusion using information at days 7, 14, 21, and 28. (Figure 2d) Patients missing either time-to-peak expansion (defined as days 7 and 14) or one timepoint following peak expansion were excluded from the analysis due to the potential bias the patterns of missingness could have on the AUC value. AUCs were calculated using the linear trapezoidal method on raw values and the results were then transformed using a base 10 logarithm. The violin plots in figure 2 shows not only the distribution of the AUCs by treatment group but the density of the data. Median values of the log-transformed AUC are displayed and to test whether AUCs differed between treatment groups, a Mann-Whitney U test was performed. The analytic dataset included 85 datapoints across 23 patients, 6 within the Bendamustine group and 17 within the Flu/Cy group.

Supplemental Results

Table S2: Baseline Characteristics

	**Bendamustine N=14**	**Fludarabine/ Cyclophosphamide, N=42**	**P-value***
	**N (%) or median (IQR)**	**N (%) or median (IQR)**	
Age	66 years (57-69)	64 years (59-69)	0.93
Sex, female	7 (50%)	18 (43%)	0.88
Race<br>Black<br>Asian<br>White<br>Other	 2 (14%)<br>1 (7%)<br>11 (79%)<br>0	 5 (12%)<br>5 (12%)<br>27 (64%)<br>5 (12%)	0.52
Extramedullary disease	9 (64%)	24 (57%)	0.88
ISS stage I, II, III, missing	10/3/1<br>(71%, 21%, 7%)	26,7,7,2<br>(62%, 17%, 17%, 5%)	0.66
High-risk cytogenetics**<br>Missing	6 (43%)	25 (60%)<br>N=1	0.42
Prior lines of therapy	5 (4-7)	6 (5-8)	0.3
Triple Refractory Disease	11 (79%)	36 (86%)	0.84
Penta Refractory Disease	7 (50%)	21(50%)	1
Bridging Therapy	10 (71%)	35 (83%)	0.56
Type of CAR-T Therapy<br>Ide-cel<br>Cilta-cel	 6 (43%)<br>8 (57%)	 30 (71%)<br>12 (29%)	0.11
Cell dose in million CAR-T cells<br>Ide-cel dose<br>Cilta-cel dose	 403 million (350-421)<br>0.6 million/kg (0.6-0.0.6)	 401 million (377-432)<br>0.6 million/kg (0.5-0.6)	 1<br>0.68

*P-values are presented before covariate balancing to fully understand the differences between treatment groups. **High risk cytogenetics were defined as presence of deletion 17p, t(4;14), t(14;16), t(14;20) and gain 1q.

Table S3: Hematologic parameters from lymphodepletion to one month post CAR-T based on bendamustine vs fludarabine/cyclophosphamide lymphodepletion

	Bendamustine N=14	Fludarabine/ Cyclophosphamide, N=42	P-value
	**median (IQR)**	**median (IQR)**	
**Absolute Lymphocyte Count, 10^9/L**			
Pre-Lymphodepletion	0.68 (0.45 – 0.98)	0.57 (0.39 – 1.08)	0.97
Day 0	0.15 (0.08 – 0.26)	0.02 (0.00 – 0.06)	**0.02**
Day 7	0.21 (0.17 – 0.47)	0.30 (0.10 – 0.60)	0.35
Day 14	0.71 (0.52 – 0.90)	0.50 (0.30 – 0.86)	0.51
Day 30	0.43 (0.39 – 0.52)	0.45 (0.27 – 1.10)	0.54
Nadir ALC count days 0-7	0.10 (0.04 – 0.17)	0.01 (0.00 – 0.02)	**<0.01**
Day for nadir ALC count for days 0-7	1.50 (1.00 – 2.75)	1.00 (0.00 – 2.00)	0.25
Nadir ALC count for days 0-30	0.09 (0.04 – 0.15)	0.01 (0.00 – 0.03)	**<0.01**
Day 60	0.71 (0.49 – 0.96)	0.82 (0.27 – 1.40)	0.81
Day 90	0.91 (0.57 – 1.06)	0.79 (0.45 – 0.98)	0.54
**Absolute Neutrophil Count, 10^9/L**			
Pre-LD	2.42 (2.17 – 3.13)	2.40 (1.80 – 3.24)	0.31
Day 0	1.57 (1.42 – 2.02)	1.46 (0.80 – 1.83)	0.24
Day 7	1.83 (1.57 – 2.07)	1.22 (0.60 – 2.30)	**0.03**
Day 14	1.82 (1.42 – 2.99)	1.80 (1.10 – 2.39)	0.16
Day 30	1.05 (0.70 – 1.41)	1.80 (1.02 – 2.58)	**<0.01**
Nadir neutrophil count days 0-30	0.90 (0.62 – 1.21)	0.34 (0.16 – 0.52)	**<0.01**
Day 60	2.92 (1.56 – 3.21)	2.10 (1.45 – 3.38)	0.65
Day 90	2.43 (1.51 – 3.05)	2.82 (1.70 – 3.82)	0.23
**Platelet count, 10^9/L**			
Pre-LD	182.50 (129.75 – 230.00)	150.50 (111.25 – 204.50)	0.96
Day 0	143.00 (117.25 – 167.75)	112.50 (75.75 – 161.50)	0.67
Day 7	128.50 (100.50 – 153.75)	66.50 (35.75 – 117.75)	0.07
Day 14	97.00 (49.00 – 161.75)	84.50 (43.00 – 154.75)	0.69
Day 30	76.50 (45.50 – 123.25)	66.00 (36.25 – 110.25)	0.96
Nadir platelet count, Days 0-30	55.50 (35.75 – 108.75)	41.50 (18.25 – 66.00)	0.68
Day 60	208.00 (133.25 – 259.75)	108.00 (54.50 – 169.00)	0.30
Day 90	186.50 (118.25 – 235.00)	117.00 (63.00 – 198.00)	0.08

**Hemoglobin, g/dL**			
Pre-LD	10.10 (9.33 – 11.20)	10.10 (9.03 – 11.68)	0.38
Day 0	9.00 (8.35 – 9.83)	8.95 (8.13 – 10.58)	0.16
Day 7	9.10 (8.25 – 10.13)	8.60 (7.60 – 9.75)	0.83
Day 14	8.80 (8.40 – 10.08)	9.55 (8.73 – 11.28)	**0.03**
Day 30	10.25 (9.73 – 10.90)	10.00 (8.30 – 11.48)	0.87
Nadir hemoglobin, Days 0-30	8.15 (7.53 – 8.83)	7.55 (6.80 – 9.02)	0.99
Day 60	10.70 (10.28 – 11.75)	10.90 (8.75 – 12.25)	**0.02**
Day 90	10.95 (10.50 – 12.30)	10.50 (9.00 – 12.90)	0.30

Table S4: Safety outcomes post BCMA CAR-T therapy based on bendamustine vs fludarabine/cyclophosphamide lymphodepletion

	**Bendamustine N=14**	**Fludarabine/ Cyclophosphamide, N=42**	**P-value**
	**N(%) or median (IQR)**	**N(%) or median (IQR)**	
CRS, any grade	11 (79%)	36 (86%)*	0.85
CRS grade $\geq$ 2	4 (29%)	6 (14%)	0.15
CRS grade $\geq$ 3	0	0	
Median time to onset of CRS<br>Ide-cel<br>Cilta-cel	 0.5 day (0-1)<br>8 days (6.5-11)	 1 day (1-1.3)<br>7 days (6-9.8)	0.45
ICANS, yes<br>Grade 1<br>Grade 2<br>Grade 3<br>Grade 4	4 (29%)<br>2 (14%)<br>2 (14%)<br>0 (0%)<br>0 (0%)	6 (14%)<br>4 (10%)<br>0 (0%)<br>1(2.4%)<br>1 (2.4%)	0.09
ICU admission	0 (0%)	1 (2%)	0.34
Neutropenic fever in first 30 days	1 (7%)	15 (36%)	0.37
Tocilizumab use	8 (57%)	26 (62%)	>0.99
Steroid use	6 (43%)	11 (26%)	0.11
IVIG use	4 (33%)	19 (29%)	0.65
PRBC transfusion needed	1 (7%)	18 (43%)	0.15
Platelet transfusion needed	0 (0%)	14 (33%)	**<0.001**
Hospital Readmission	2 (14%)	5 (12%)	0.44
G-CSF use	10 (71%)	39 (93%)	0.10
Infection	3 (21%)	15 (35%)	0.57
**Grade ≥ 3 cytopenia at Day 30**			
Grade ≥ 3 neutropenia	6 (43%)	12 (29%)	**0.048**
Grade ≥ 3 anemia	0 (0%)	9 (22%)	**0.004**
Grade ≥ 3 thrombocytopenia	6 (43%)	16 (38%)	0.14

Abbreviations: ALC: Absolute lymphocyte count, ANC: Absolute neutrophil count, CRS: Cytokine release syndrome, ICANS: Immune effector cell associated neurotoxicity syndrome; ICU: Intensive care unit; LD: Lymphodepletion; PRBC: Packed red blood cells.
*CRS grade was not quantifiable in one patient in the Flu/Cy group.

For binary and categorical variables, weighted chi square tests were implemented. For continuous outcomes, Mann-Whitney U tests were chosen as a nonparametric alternative to t-tests. For time-to-event outcomes (i.e., time-to-onset of CRS or ICANS), a log rank test was used. The IPTW variable was included as a weighting variable for all inferential tests.

Table S5: Safety and Efficacy outcomes Based on Type of BCMA CAR-T therapy and bendamustine vs fludarabine/cyclophosphamide Lymphodepletion

	Cilta-cel		Ide-cel		P-value*
	**Bendamustine**	**Flu/Cy**	**Bendamustine**	**Flu/Cy**	
CRS grade ≥ 2	3 (38%)	1 (8%)	1 (17%)	5 (17%)	0.16
Median time to CRS onset	8 days	7 days	0.5 days	1 day	0.48
ICANS Any grade	3 (38%)	1 (8%)	1 (17%)	5 (17%)	0.11
Median time to ICANS onset	6 days	5 days	5 days	1 day	0.06
Grade ≥ 3 Neutropenia	5 (62%)	4 (33%)	1 (17%)	8 (27%)	0.18
Grade ≥3 Anemia	0 (0%)	1 (9%)	0 (0%)	8 (27%)	0.29
Tocilizumab use	5 (62%)	4 (33%)	3 (50%)	22 (73%)	0.76
Steroid use	5 (62%)	1 (8%)	1 (17%)	10 (33%)	0.15
IVIG use	3 (38%)	3 (25%)	1 (25%)	7 (32%)	0.70
GCSF use	6 (75%)	10 (83%)	4 (67%)	29 (97%)	0.15
ORR*	7 (88%)	10 (83%)	4 (67%)	23 (88%)	0.60
CR*	4 (50%)	6 (50%)	4 (67%)	10 (38%)	0.52
PFS	6-month: 63% (95% CI: 23%-86%)	6-month: 65% (95% CI: 31%-85%)	Median: 7.7 months (95% CI: 3.4 - NR)	Median: 7.6 months (95% CI: 6.2 – NR)	0.99**
OS	6-month: 86% (95% CI: 33%-98%)	6-month: 73% (95% CI: 37%-91%)	6-month: 100% (95% CI: NR- NR)	6-month: 87% (95% CI: 68% - 95%)	0.11**

Overall, 4 patents had non-measurable disease by IMWG crtieria and are excluded from response analysis, all from the Flu/Cy group. Note: P-values are derived from multivariable logistic regression for given variables comparing bendamustine vs Flu/Cy lymphodepletion in the IPTW sample after adjusting for CAR-T type** P-values are derived from multivariable Cox proportional hazards analysis for PFS and OS in the IPTW sample after adjusting for CAR-T type

Figure S2: Overall survival Based on Type of BCMA CAR-T therapy and Bendamustine vs Fludarabine/Cyclophosphamide Lymphodepletion (a) Cilta-cel and (b) Ide-cel

a.

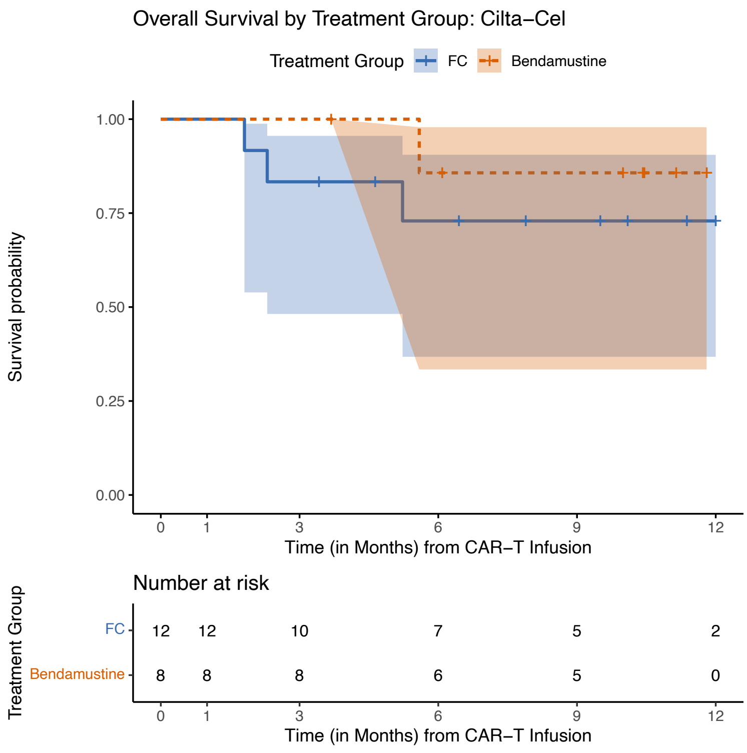

b

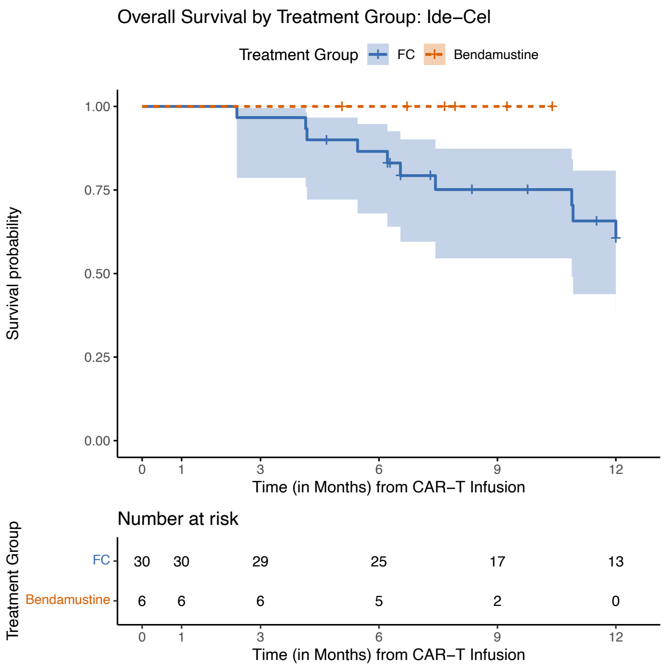

Supplement: Supplementary file 1 — Supplementary Data [file 41408_2023_929_MOESM1_ESM.pdf]
